# Synergism of Chinese Herbal Medicine: Illustrated by Danshen Compound

**DOI:** 10.1155/2016/7279361

**Published:** 2016-04-13

**Authors:** Xuefeng Su, Zhuoting Yao, Shengting Li, He Sun

**Affiliations:** Department of Clinical Research, Tasly Pharmaceuticals Inc., 9400 Key West Avenue, Rockville, MD 20850, USA

## Abstract

The primary therapeutic effects of Chinese herbal medicine (CHM) are based on the properties of each herb and the strategic combination of herbs in formulae. The herbal formulae are constructed according to Chinese medicine theory: the “Traditional Principles for Constructing Chinese Herbal Medicinal Formulae” and the “Principles of Combining Medicinal Substances.” These principles of formulation detail how and why multiple medicinal herbs with different properties are combined together into a single formula. However, the concept of herbal synergism in CHM still remains a mystery due to lack of scientific data and modern assessment methods. The Compound Danshen Formula (CDF) is a validated formula that has been used to treat a variety of diseases for hundreds of years in China and other countries. The CDF will be employed to illustrate the theory and principle of Chinese herbal medicine formulation. The aim of this review is to describe how Chinese herbal medicinal formulae are constructed according to Chinese medicine theory and to illustrate with scientific evidence how Chinese herbs work synergistically within a formula, thereby supporting Chinese medicine theory and practice.

## 1. Introduction

Traditional Chinese Medicine (TCM) encompasses a variety of therapies including Chinese herbal medicine (CHM), acupuncture, Qigong, and physical therapy such as massage and Gua Sha (scraping). Due to its unique philosophy and treatment characteristics, TCM was identified as one of the advanced medical sciences until the 17th century [1, 2]. CHM, as the most important part of TCM, has been used to treat disease for over 4,000 years [1, 3]. Even though Western medicine is widely accepted by modern Chinese culture, CHM still plays a very important role in daily medical practice; about 46% of patients were prescribed CHM in hospitals or outpatient clinics. CHM is popularly used for the treatment of variety of diseases such as the common cold, hepatitis, nephrotic syndrome, diabetes, cardiovascular disease, and cancer [4–9]. Worldwide, increasing numbers of Westerners are recognizing the importance of CHM and seeking it as part of their healthcare.

Medical science has long realized that the pathogenesis and progression of diseases are too complex for single drug treatment. For example, cardiovascular disease is the primary killer worldwide [10, 11], with coronary artery disease (CAD) as the most common cause of cardiovascular morbidity and mortality. The pathophysiology of CAD is very complex and warrants multifaceted treatment [12, 13]. The strategies include treatment of the primary disease as well as intervention in risk factors and comorbid conditions such as high cholesterol, hypertension, obesity, and diabetes [14, 15]. The treatment regimen is a combination of pharmaceutical drugs that target the different organs and systems involved in the pathophysiological processes of the primary and secondary conditions. These medications include but are not limited to aspirin, nitrates, Angiotensin II receptor blocker (ARB), statins, diuretics, and metformin [15–17]. Interestingly, to achieve better therapeutic outcomes and diminish side effects, multiherb therapy is an essential component of traditional Chinese herbal medicine and has been utilized in China for thousands of years [3, 18].

Originally, practitioners of Chinese herbal medicine used just single herbs for disease treatment. But, over time, Chinese herbalists gained more experience from clinical practice and learned that the causes of diseases were imbalances among different systems in the body. To restore the dynamic balance among body systems and get the best curative effect with the least toxicological effect, Chinese herbalists chose a combination of several herbs based on the distinct disease presentations. Through this process, the theory of herbal synergism in CHM was developed and refined by herbalists over thousands of years of clinical practice. Single herbal medicines and herbal formulae compose the Chinese herbal medicine system [19]. There are 8980 herbs compiled into* Zhong Hua Ben Cao* (*Chinese Materia Medica* 1999) and 1444 Chinese herbal formulae collected in 2010 edition of the* Chinese Pharmacopoeia*. CHM combines two or more herbs with different properties synergistically to form a compound formula named “复方 (fù fānɡ)” or “方剂 (fānɡ jì)” based on the Principles of Combining Medicinal Substances [20].

When two or more Chinese herbal medicines (CHMs) are combined, they become a named formula. Over 2,000 years of practice, CHM has accumulated over 100,000 formulae that are constructed according to specific TCM principles [18, 21]. Based on empirical evidence gathered over many centuries, CHM providers learned that particular herbal combinations work synergistically or antagonistically [3]. Herbal formulae are constructed based on this synergism, outlined as theories in* Materia Medica*. With the rise of scientific research methods, the synergism between herbs is being investigated pharmacologically. This research substantiates Chinese medicine theory and elucidates correct dosage combinations [22]. Through the example of a cardiovascular herbal formula, this paper will explain how Chinese herbs are combined into formulae, the seven traditional types of herbal combinations, and how scientific research contributes additional knowledge.

## 2. Traditional Principles for Constructing Chinese Herbal Medicinal Formulae: Seven Types of Herbal Combinations

Traditionally, there are seven types of Chinese herbal combinations, differentiated by their physiologic effects (seven emotions, Chinese name 七情 qīqínɡ). Seven emotions have two different definitions in TCM. The first one emphasizes the relation between diseases and mental activities, the main pathogenic factors of endogenous diseases [23]. Emotional mental activities are categorized as the seven emotional factors: joy, anger, melancholy, worry, grief, fear, and fright. This is beyond the scope of our topic.

The second one is used in herbal medicine to elucidate the seven possible outcomes that could be yielded if multiple herbs with different pharmacological properties are used in combination. It was first recorded in* Shennong Bencao Jing*, a classic book systemically describing CHM that was written approximately 2000 years ago between the Qin and Han dynasties. From a modern pharmacology perspective, this is a theory about herb-herb interactions [24, 25]. These were described in the* Grand Materia Medica*.

*(1) Single Effect (单*行* dān xín*ɡ). Single effect (单行 dān xínɡ) is the use of one medicinal substance to treat a patient. An example is the Du Shen Tang decoction, which consists of only one herbal medicine,* Panax ginseng* (Asian ginseng). It was historically prescribed to patients with “Qi and Xue deficiency” states such as postpartum hemorrhage [26], acute myocardial infarction [27], cirrhosis [28], upper gastrointestinal bleeding [29], and congestive heart failure [30].

*(2) Mutual Accentuation (*相*須 xiāng xū)*. Mutual accentuation (相須 xiāng xū) means that the combination of two herbal substances with similar functions will accentuate their therapeutic actions, also called “mutual necessity.” For instance, herbal decoction of Da Huang (*Rheum palmatum*, rhubarb) and Mang Xiao (*natrii sulfas*, mirabilite) is used to treat constipation. Pharmacological research showed that Da Huang is able to stimulate the colon by increasing the intensity and frequency of contractions, which subsequently improves bowel motility. The Mang Xiao contains sodium sulfate, which when absorbed in colon creates a hyperosmotic environment, bringing water into the colon and softening the stool. When Da Huang and Mang Xiao are used together in combination they have a better laxative effect compared to each used alone [25, 31].

*(3) Mutual Enhancement (*相*使 xiāng shǐ)*. Mutual enhancement (相使 xiāng shǐ) is the combination of two or more substances with different actions in which one of the substances enhances the effect of the other in a specific clinical situation, also called “mutual employment.” For example, Huang Lian (*rhizoma coptidis*, coptis root) and Mu Xiang (*vladimiriae radix*, costus root) are herbs often prescribed as a pair for the treatment of dysentery. Modern pharmacological study has demonstrated that Huang Lian has antimicrobial effect against* Shigella* spp. both* in vivo* and* in vitro*. Mu Xiang has been proven to increase the serum concentration of berberine, the major active antimicrobial component of Huang Lian. When used with Mu Xiang, Huang Lian has a greater efficacy for patients with dysentery [25, 32].

*(4) Mutual Counteraction (*相*畏 xiāng wèi)*. Mutual counteraction (相畏 xiāng wèi) literally translates to “mutual fear” and means a combination in which the toxicity or side effects of one substance are reduced or eliminated by another substance. For instance, Fu Zi (*Aconitum carmichaelii*, aconite root) is a toxic herb where the primary component is harmful to cardiac and nerve cells. When it is prepared with Gan Cao (*radix glycyrrhizae*, licorice root), the toxicity of Fu Zi is diminished [25, 33]. The emphasis in mutual counteraction is on the toxic herb that is being counteracted in the formulation.

*(5) Mutual Suppression (*相*杀 xiāng shā)*. Mutual suppression (相杀 xiāng shā) literally translates to “mutual killing” and is the reverse of mutual counteraction. With mutual suppression, one substance also reduces the undesirable side effects of another but the emphasis is on the herb that performs the beneficial suppressive action. For example, Sheng Jiang (*Zingiber officinale*, ginger root), which was commonly prescribed for cold prevention, as antiemetic, and for detoxication, suppresses or literally “kills” the toxicity of Ban Xia (*Pinellia ternata*, pinellia root) commonly used to relieve cough and to stop vomiting and it is toxic when used alone. In short, mutual counteraction and mutual suppression describe the same herb-herb interaction, but from differing viewpoints [25, 33].

*(6) Mutual Antagonism (*相*恶 xiāng wù)*. Mutual antagonism (相恶 xiāng wù) literally translates to “mutual aversion” and means the ability of two substances to minimize each herb's positive effects. Traditionally, there are eight pairs and one trio of substances that have mutually antagonistic effects on each other. Together they are referred to as the “nineteen antagonisms (十*九畏* shí jiǔ wèi) [34].” Ren Shen (*radix ginseng*, Chinese ginseng) is capable of replenishing Qi, which increases the overall function of the body. This beneficial function can be reduced or abolished if combined with the herb Lai Fu Zi (*semen raphani*, radish seed) [25, 33].

*(7) Mutual Incompatibility (*相*反 xiāng fǎn)*. Mutual incompatibility (相反 xiāng fǎn) translates literally to “mutual opposition” and occurs when the combination of two substances causes side effects or toxicity which would not be caused by any one of the substances if used alone. Traditionally there are three sets or a total of eighteen substances called the “eighteen incompatibilities' (十*八反* shí bā fǎn) [35].” For example, it is prohibited in CHM to use Danshen (*Salvia miltiorrhiza*, red sage) and Li Lu (*Veratrum nigrum*, black false hellebore) together. A toxicological study showed that when Danshen was combined with Li Lu, the Danshen increased the concentration of harmful* Veratrum* alkaloids that can produce serious adverse effects such as nausea, vomiting, dizziness, hypotension, and tachycardia. Thus, such a mutually incompatible pair of herbs should not be used in clinical practice according to the principles of CHM [25, 35].

## 3. The Principles of Combining Medicinal Substances: King, Ministers, Adjutants, and Messengers (***君臣佐使*** jūn chén zuǒ shǐ)

The Principles of Combining Medicinal Substances provide guidelines for the composition of Chinese herbal prescriptions. Traditionally described in terms of a feudal hierarchy, a formula is usually composed of several medicinal herbs [22, 25]. The chief (also called king, sovereign, or lord, in Chinese 君 jūn) is the principal herbal ingredient in modern texts and is the substance that provides the main therapeutic effect in the prescription. The deputies (also called ministers or associates, in Chinese 臣 chén) enhance or assist the therapeutic actions of the chief. The assistants (also called adjutants, in Chinese 佐 zuǒ) provide one or more of the following functions: treating accompanying symptoms, moderating the harshness or toxicity of the primary substances, assisting the chief and deputies in accomplishing their main objectives, or providing assistance from another therapeutic direction, such as the addition of cooling substance to a warming prescription or vice versa. The envoys (also called messengers or couriers, in Chinese 使 shǐ) either guide the other herbs in the formula to a specific channel or organ or exert a harmonizing influence. A common herb used as a messenger is glycyrrhizae root (*甘草* gān cǎo). Not all principles need to be used in every herbal prescription. There are many simple prescriptions that contain only a chief and deputies, and there are prescriptions in which one substance serves more than one function. For instance, there may be multiple adjutants and messengers in one formula, and one herb may act both as an adjutant and as a messenger [36, 37].

**Figure 1:**
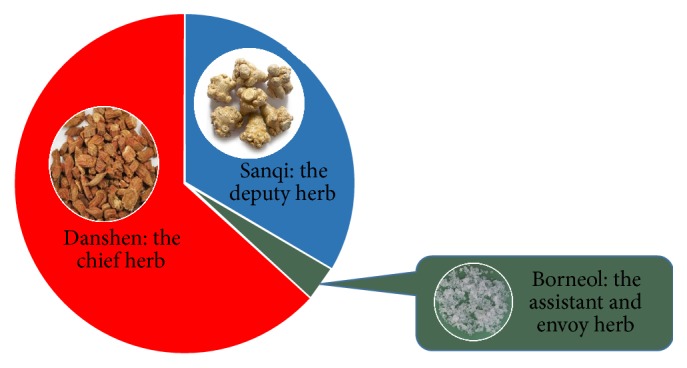
The illustration of the role of each ingredient in Fufang Danshen formula. In this formula, Danshen serves as the chief herb and the principal medicine. While Sanqi serves as the deputy herb, enhancing the therapeutic action of Danshen. Bingpian is the assistant and envoy herb that increases the blood concentration of Danshen and Sanqi.

A good example to illustrate the different roles of herbs in formulation is the Compound Danshen Formula (Figure 1), a commonly used formula for treating coronary artery disease including angina and acute myocardial infarction [9]. In this formula, Danshen (*Salvia miltiorrhiza*, red sage) serves as the chief herb. Pharmacological research shows that Danshen causes relaxation of the coronary arteries which is one of the herb's major physiologic, cardioprotective effects. Another herb, Sanqi (*Panax notoginseng*, notoginseng), serves as the deputy herb in the formulation because it has its cardiomyocyte protective and antiplatelet functions. A third herb, Bingpian (*Dryobalanops aromitaca*, borneol), serves as assistant and envoy by increasing the blood concentration of Danshen and Sanqi [38, 39].

## 4. Pharmacological Research on the Synergism among* Danshen*,* Sanqi*, and* Bingpian*

Compound Danshen Formula (CDF), composed of Danshen, Sanqi, and Bingpian, has been widely used in the treatment of cardiovascular diseases in China and other countries for over thirty years [40–42]. CDF has been employed by over 600 Chinese pharmaceutical companies and many different products were produced from CDF in China [43].

The dried root of plant Danshen is a popular herbal medicine in China and Japan, used alone or in combination with other herbs [44, 45]. It was first recorded in the Shennong's Classic Materia Medica,* Shennong Bencao Jing*, which is the oldest medicine monograph in China. One of the most widely used traditional medicines, Danshen, is used in the treatment of coronary heart disease [46], cerebrovascular disease [47], Alzheimer's disease [48], Parkinson's disease [49], renal deficiency [50], liver cirrhosis [51], cancer [52], and bone loss [53]. The composition of Danshen has been analyzed and found to contain 49 diterpenoid quinones, 36 hydrophilic phenolic acids, and 23 essential oil constituents. The diterpenoid quinones and hydrophilic phenolic acids are the principal bioactive components in Danshen [54].

The dry root of Sanqi has been traditionally used in CHM for thousands of years as a hemostatic medicine to control internal and external bleeding [55]. Currently, Sanqi is a commonly used herb to treat cardiovascular disease by stopping bleeding, as well as invigorating and supplementing blood [56]. Moreover, it has function of protecting myocardium, specifically for improving ischemia/reperfusion (I/R) induced injury after percutaneous coronary interventional therapy [54, 57]. It has been reported to have antihypertensive, antithrombotic, antiatherosclerotic, and neuroprotective activities [57]. Various chemical constituents in Sangi have been identified, including ginsenosides, notoginsenosides, flavonoids, volatile oils, amino acids, and polysaccharides [58].

Bingpian contains monoterpenoid constituents and is included in 63 CHM prescriptions according to the* Chinese Pharmacopoeia*. Its wide use as an assistant in CHM is due to its ability to improve percutaneous absorption, enhance oral bioavailability, and facilitate passage of herbal formulation through the blood brain barrier [59–61].

It has been demonstrated through years of clinical use that Danshen and Sanqi have a synergistic effect when combined but the molecular mechanisms underlying their synergism have yet to be clearly elucidated [41, 62]. Cardiovascular disease is the number one cause of death in Europe and in the United States according to the Centers for Disease Control in 2013 [10, 63]. Compound Danshen Formula (CDF) has been widely accepted and used in the treatment of cardiovascular diseases in China and other countries for decades with beneficial outcomes confirmed by clinical trials [9, 64]. There are three preparations compiled in the* Chinese Pharmacopoeia 2010 edition*: Compound Danshen Tablet (CDT), Compound Danshen Granule (CDG), and Compound Danshen Dripping Pill (CDDP). In 1997 the United States Food and Drug Administration (FDA) accepted the Chinese medicinal product CDDP as an investigational new drug (IND number 56956) [9, 64]. In 2010, a phase II clinical study on CDDP was completed and now a phase III clinical trial is underway (ClinicalTrials.gov Identifier: NCT01659580), which is an important milestone in the incorporation of CHM into the Western medical system.

Effects of Danshen and Sanqi on the cardiovascular system are summarized as follows: (1) antioxidant and cardiac protection [65, 66]; (2) inhibition of platelet aggregation and adhesion [67, 68]; (3) vasorelaxation [69]; and (4) prevention of arthrosclerosis [70]. Sanqi prevents arthrosclerosis [41, 71] and protects cardiac myocytes [65, 72]. Danshen contains both hydrophobic and hydrophilic bioactive components such as danshensu, salvianolic acid B, and tanshinone [73–75]. Sanqi contains abundant saponins such as ginsenosides and notoginsenosides [76]. When the two herbs are decocted together in a combination formula, studies have shown that the interaction between the herbs results in a compound with stronger pharmacological properties compared to each herb alone [62].

### 4.1. Synergistic Effect of Danshen and Sanqi Demonstrated by Chemistry and Pharmacokinetic Studies

Zeng et al. [77] performed an* in vitro* comparative study on the influence of Danshen and Sanqi on the dissolution of active components from Danshen when the two herbs were decocted in different mass ratios. The ethanol extracts from Danshen and Sanqi were decocted in different mass ratio and HPLC was employed to quantitate the active components from Danshen. The result demonstrated that the main active ingredients from Danshen is higher in all codecocted groups than in the Danshen only group with highest extraction rate at ratio of 5 : 3, Danshen to Sanqi, which is the ratio in classic CDF. In contrast, the effect of mixture or single decoction of Danshen and Sanqi on the dissolution of active components from Sanqi differs from that of Danshen. The effect of Danshen and Sanqi on the extraction of active components from Sanqi was studied by using HPLC combined with UV-visible spectroscopy, infrared spectroscopy, and time-of-flight mass spectrometry [78]. The results demonstrated that codecoction of Danshen and Sanqi inhibited the dissolution of the active components from Sanqi.

A recent* in vivo* study on Guinea pigs extended these findings using three experimental groups [79]. Danshen extracts (salvianolic acid B and tanshinone IIA), Sanqi extracts (panax notoginseng saponins), and a combination of the two extracts were given to three groups of Guinea pigs for a pharmacokinetic study. Parameters analyzed included maximum blood concentration (*C*_max_) and area under curve [(AUC) (0–*t*)]. The result demonstrated that the pharmacokinetic levels of active constituents salvianolic acid B (SalB), tanshinone IIA (Ts IIA), notoginsenoside R1 (R1), ginsenoside Rg1 (Rg1), and ginsenoside Rb1 (Rb1) were markedly different in the Danshen-Sanqi combination group compared to the groups that took Danshen and Sanqi alone. *C*_max_ of R1, Rb1, and Rg1 were significantly decreased while *C*_max_ of Ts IIA and SalB were increased in both cerebrospinal fluid (CSF) and plasma. These effects are most likely achieved by increased absorption and distribution of the compound in the body, a result of coadministration of Danshen and Sanqi. This study shows the significant pharmacokinetic changes that can result from the herb-herb interactions. Another study that showed similar results administered oral Danshen alone, Sanqi alone, or Danshen and Sanqi combination suspension to beagle dogs [80]. After administration, the plasma concentration-time profiles of danshensu, tanshinone IIA, cryptotanshinone, notoginsenoside R1, ginsenoside Rg1, and ginsenoside Rb1 were analyzed by LC-MS/MS. The results showed that both *C*_max_ and AUC of Danshensu, notoginsenoside R1, ginsenoside Rg1, and ginsenoside Rb1 in the Danshen and Sanqi combination group decreased in comparison with those in either the Danshen alone or Sanqi alone groups.

### 4.2. Synergistic Interaction and Cardiac Protection of Danshen and Sanqi in an Acute Myocardial Infarction Animal Model

Pharmacological studies indicate that the coadministration of Danshen and Sanqi has a cardiac protective effect of improving coronary circulation and improving symptoms of myocardial ischemia, while individual administration of Danshen expands blood vessels and individual administration of Sanqi targets cardiac myocytes. Specifically, the herb pair exerts the best cardiac protective effects when the mass ratio of Danshen to Sanqi is between 10 : 3 and 10 : 7 [41, 81]. A preclinical study [82] comparing the cardioprotective effect of salvianolic acid (SAL) and tanshinone (TAN) was performed in a rat model of acute myocardial infarction (MI). Rats were randomly assigned to four groups: sham group (the ligation suture was placed in the heart, but without ligation), myocardial infarction (MI) group, SAL treatment group, or TAN treatment group (SAL + MI, TAN + MI, resp.). The MI was produced by occlusion of the left anterior descending coronary artery and SAL (120 mg/kg) and TA (120 mg/kg) were given once daily after MI by oral administration. The cardiac functional parameters were measured at 3, 7, 14, and 28 days after surgery. The data demonstrated that both the SAL and TAN treatment groups delayed the development of ischemia by decreasing infarct size and improving systolic function after MI. Gene chip analysis indicated different kinetics and gene expression profiles presented after the administration of SAL and TAN. SAL acted in a later period after ischemia, and its effect was likely mediated by downregulation of genes involved in oxidative stress, specific G-protein coupled receptor activities, and apoptosis [82]. Meanwhile TAN acted relatively early after ischemic injury and its effect was mediated by the inhibition of intracellular calcium, cell adhesion, and alternative complement pathway. Although both SAL and TAN contributed to the cardioprotective effect of Danshen, there were significant mechanistic and temporal differences between the two constituents. To test the effects of the major components from Danshen and Sanqi on cardiac function in a myocardial infarction (MI) rat model, salvianolic B (SalB) from Danshen and ginsenosides Rg1 (GRg1) and Rb1 (GRb1) from Sanqi were administered alone or in combination intragastrically [83]. Fifty rats were randomized into six groups: Sham operation, MI + saline, MI + SalB, MI + GRg1, MI + GRe, MI + SalB + GRg1 (mass ratio of SalB to GRg1 is 2 : 5), and MI + SalB + GRe (mass ratio of SalB to GRe is 2 : 5). The medication was administered in a 60 mg/kg dose twice. The first dose was given one hour before MI generation and the second dose was given at 23rd hour after MI. The group who received the combination of SalB and GRg1 at the mass ratio of 2 : 5 significantly improved cardiac function in this MI model, illustrated by increased left ventricular contractility (+dp/dt) parameter without negative effects on heart rate or blood pressure. No significant improvement in +dp/dt was noted in rats administrated with SalB, GRg1, or GRb1 alone. Nor was improvement seen in the group with coadministration of SalB and GRb1. This result demonstrated that SalB from Danshen and GRg1 from Sanqi worked synergistically to improve cardiac function in a myocardial infarction rat model with mass ratio of 2 : 5.

Lu et al. [84] compared the cardioprotective effects of tanshinone IIA (T), salvianolic acid B (S), ginsenoside Rb1 (G), and Compound Danshen Formula (CDF) on acute myocardial ischemia in rats. The T, S, G, TSG (combination of T, S, and G), and CDF were administered to the MI rats. The cardioprotective effect was evaluated by measuring MI associated parameters including ECG and cardiac enzymes. The result indicated that the combination administration of TSG, and not the administration of the single components alone, had a similar beneficial cardiac effect on MI rats as the CDF group. Other cell biology studies confirmed these findings. Zeng et al. [85] reported that combinational use of Danshen and Sanqi had protective effects on human umbilical vein endothelial cells (HUVEC) that underwent hypoxia-reoxygenation induced cell injury. Measurement of LDH leakage, an index of cell injury, in a culture medium from cells treated by the mixture of Danshen and Sanqi prepared in different ratios demonstrated that Danshen and Sanqi had protective effects on HUVEC at the ratios of 10 : 1, 5 : 3, 1 : 1, and 0 : 10, with best benefit effect at ratios of 5 : 3 and 1 : 1, Danshen to Sanqi.

### 4.3. Combination of Danshen and Sanqi in the Modulation of Platelet Function

Herbal combination of Danshen and Sanqi in different mass ratios (10 : 0, 10 : 1, 10 : 3, 10 : 6, and 1 : 10, Danshen to Sanqi) significantly inhibited ADP-induced platelet aggregation with the best inhibitory action at 10 : 3, although Sanqi alone did inhibit platelet aggregation [86]. Three combinations at ratios of 10 : 3, 10 : 6, and 0 : 10 had the ability of inhibiting platelet adhesion with the best result at 0 : 10, that is, Sanqi alone. Interestingly, the mass combination of Danshen and Sanqi at ratios of 10 : 0, 10 : 1, and 1 : 10 did not affect platelet adhesion. Another* in vivo* study showed that administration of total salvianolic acids (TSA) extracted from Danshen and total notoginsenosides (TNG) from Sanqi at a dose of 550 mg/kg/day for five days could significantly inhibit ADP-induced platelet aggregation [87]. Moreover, even though the use of TSA or TNG alone and the combination of TSA and TNG at mass ratios of 1 : 1, 1 : 5, and 5 : 1 all showed significant inhibition of platelet aggregation, the combination of TSA and TNG at a ratio of 5 : 1 had the best synergistic effect on platelet aggregation. However, there is no synergistic effect on platelet aggregation of the combination of Danshen and Sanqi* in vitro* [84]. High performance liquid chromatography analysis of the plasma of rats that received TSA, TNG, or combination of TSA and TNG showed that coadministration of TNG caused change in the plasma distribution profile of TSA. The influence of combination on the absorption and/or metabolism of SA may be one of the reasons for the synergism of TSA and TNG* in vivo*.

The above-mentioned research results suggest that Danshen-Sanqi herb pair exerts multitarget and multifunction effects that single herb usage could not achieve. Additionally the research shows that a specific herbal combination ratio is critical for maximum synergetic action of Danshen and Sanqi.

### 4.4. The Role of Bingpian in Compound Danshen Formula

To define the role of Bingpian in Compound Danshen Formula (CDF), a pharmacokinetic study was conducted in rats [38]. After oral administration of extract from Danshen alone or Danshen extracts combined with Bingpian, plasma concentrations of rosmarinic acid (RA), salvianolic acid A (SAA), and salvianolic acid B (SAB) were assessed at different time points. In comparison with Danshen extracts alone, significant changes in pharmacokinetic parameters of RA, SAA, and SAB were observed in the Danshen-Bingpian group. The bioavailability of all three salvianolic acids increased when the Danshen extracts and Bingpian were administrated together. These results indicated that Bingpian could enhance intestinal absorption, decrease distribution in the body, and inhibit the metabolism of salvianolic acids. Coadministration of salvianolic acid B, saponins, and Bingpian upregulated mRNA of vascular endothelial growth factor (VEGF) in a rat model with focal cerebral ischemia/reperfusion injury [39]. In comparison with rabbits given Sanqi extract alone, animals simultaneously taking Sanqi extract and Bingpian exhibited significant differences in pharmacokinetic parameters of notoginsenosides R1 (NGR1), ginsenosides Rg1 (GRg1), and Re (GRe), which are three major active components of Sanqi. The plasma concentration of NGR1, GRg1, and GRe is increased significantly in rabbits given simultaneously Sanqi extract and Bingpian via improvements in their absorption and bioavailability. For Bingpian combined with Sanqi extract, the three saponin levels were all increased markedly in heart, lung, liver, and brain tissues with peak levels at one hour after the administration of Sanqi extract [88].

This data indicates that, in the Compound Danshen Formula, Bingpian acts as adjutant and messenger herb and can increase the blood concentration of Danshen and Sanqi.

### 4.5. Bioactive Equivalent Combinatorial Components (BECCs) Study: The Example of Synergistic Interaction of Multiple Components in CDF

Even though more than 100 components have been isolated and identified in Danshen and Sanqi to date [84], only a small fraction of these components have been studied to confirm their pharmacological effects. The following study provided a new method for the evaluation of CHM synergism. A bioactive equivalent oriented feedback screening method was developed and applied to discover the bioactive equivalence of combinatorial components (BECCs) from a cardiotonic pill (CP, a CDDP formula) [89]. To obtain the components of candidate BECCs, the real-time components trapping and combining system was employed. Eighteen components were identified as BECCs by HPLC-UV chromatography including ten phenolic acids, four saponins, and four tanshinone. The subsequent* in vitro* study showed that only the combination of 18 components had the bioequivalent protective activity as the CP in cultured HUVEC. None of the combinations (phenolic acids + saponins, phenolic acids + tanshinone, or tanshinone + saponins) exerted similar cell protective effect as the CP. In an* in vivo* evaluation test, the combination of 18 components of BECCs was identified to be as effective as the CP in improving the ischemic parameters of cardiac enzymes and left ventricular function in a rat model with myocardial infarction.

### 4.6. Herb-Drug Interaction Explored by Coadministration of CDDP and Warfarin

Due to high healthcare costs, easy access to over-the-counter herbal products, and an interest in natural approaches to disease treatment, increasing numbers of patients in the USA are turning to alternative and complementary medicines [90]. In parallel, the rate of consumption of herbal products in conjunction with conventional medications has increased and with it the potential for adverse herb-drug interactions increased [91–93]. Due to Compound Danshen Formula's wide clinical use, a recent concern has been raised about interactions between various herbal products and warfarin [91]. A study was conducted to evaluate whether CDDP interacted with warfarin when administered concomitantly: the results demonstrated that CDDP in rats did not significantly alter the pharmacodynamics of warfarin [94]. It was speculated that the interactions between CDDP and warfarin was likely to be negligible [95].

## 5. Conclusion

Chinese herbal medicine (CHM) is a major part of Traditional Chinese Medicine (TCM) and the use of synergistic compound formulae (复方 fù fānɡ) is a main therapeutic tool that is customarily composed of multiple medicinal herbs with different pharmacological properties. The combinational use of herbal medicines is at the heart of CHM and continues to play a very important role in the treatment of disease. The synergism of herbs is based on the Traditional Principles for Constructing Chinese Herbal Medicinal Formulae and the Principles of Combining Medicinal Substances. The principles are evidenced by an example formula, Compound Danshen Formula, and recent research has unveiled pharmacological and pharmacokinetic properties of the formula. This review provides preliminary explanation of CHM theory, which helps to better understand the rationale and the principal mechanism of herbal synergism and the clinical application of the formulae. However, further investigation is needed to provide more evidence of the molecular and cellular mechanisms of the synergism of CHM formulae.
